# Investigation of the Dendritic Structure Influence on the Electrical and Mechanical Properties Diversification of the Continuously Casted Copper Strand

**DOI:** 10.3390/ma13235513

**Published:** 2020-12-03

**Authors:** Małgorzata Zasadzińska, Tadeusz Knych, Beata Smyrak, Paweł Strzępek

**Affiliations:** Faculty of Non-Ferrous Metals, AGH University of Science and Technology, 30-059 Krakow, Poland; tknych@agh.edu.pl (T.K.); smyrak@agh.edu.pl (B.S.); strzepek@agh.edu.pl (P.S.)

**Keywords:** Cu-ETP, dendritic structure, continuous casting, strand casting, Cu_2_O eutectic copper oxide

## Abstract

The properties of copper in its solid state are strongly affected by the crystallization conditions of the liquid material. ETP grade copper (Electrolytic Tough Pitch Copper) contains oxygen, which causes Cu_2_O oxide to crystallize in the interdendritic spaces during solidification process which due to the shape of continuous casting mould and the feed of liquid copper during the crystallization process in strand casting might cause a high risk of macrosegregation of oxygen in the copper structure. In the current paper the implied interactions of the dendritic structure of the copper strand in terms of homogeneity at the cross-section of its electrical, mechanical and plastic properties determined based on the samples taken parallelly and perpendicularly to the surface of the dendritic boundaries were analysed. The obtained results were confronted with scanning electron microscopy (SEM) images of the fractures formed during uniaxial tensile test. It has been observed that when the crystallites were arranged perpendicularly to the tensile direction the yield strength (YS) was lower and the fractures were brittle. On the other hand, when the crystallites were arranged parallelly to the tensile direction the fractures were plastic and elongated necking was observed along with the higher YS and total elongation values. The differences in values vary in terms of the applied direction of the tensile force. A characteristic positioning of the Cu_2_O oxide particles inside the fracture depending on the crystallite alignment and the direction of the applied tensile force has been observed.

## 1. Introduction

The mechanical properties such as ultimate tensile strength (UTS) and YS of the material depend on its structure, which may be controlled throughout the technological processes. However, appropriate control of the material structure is much more difficult when the processes is conducted continuously such as strand casting process of copper intended for direct processing into wire rod designated for electrical purposes (Cu-ETP). It is, in fact, a continuous casting process of copper strand directly subjected to the hot-rolling process. The strand is obtained via a mobile casting mould [1,2] and due to the symmetrical cooling of the strand surface a fast and uniform crystallization process of the liquid metal takes place. Uniform growth of nuclei in all directions during the crystallization process and the preferential directions of heat abstraction (the direction is perpendicular to the surface of the casting mould) form the dendritic structure of the strand. The resulting dendrites grow until they begin to contact each other (crystallization fronts), which inhibits their further growth. The microstructure of metallic materials formed during crystallization is called the primary structure and strongly influences the material macroscopic properties [3]. The outer layers during crystallization process solidify first forming a thin, fine-grained zone of frozen grains. Large columnar grains with preferred crystallographic alignment are formed next (in agreement with the direction of heat abstraction). The dendritic structure, due to its heterogeneity is disadvantageous and creates, among others, risk of macrosegregation of oxygen present in the copper structure and lowering the properties of the strand in the solid state [4]. This phenomenon is well known and described by Hall-Petch relation, according to which the grain size affects the YS of metals [5,6], but also the specific arrangement of the structure. The correlation between the mechanical properties and the structure of the casted materials has been investigated in numerous publications, mainly concerning alloys [7,8,9,10,11], and it has been observed that both are strictly related to each other. It is especially observable when the casted material consists of a columnar structure and the crystallites are specifically aligned causing the properties obtained in the uniaxial tensile test to become anisotropic [3]. An important factor concerning ETP grade copper is also the presence of Cu_2_O eutectic oxide located in interdendritic spaces and may significantly affect the mechanical and plastic properties of the material [12,13]. Regarding the ETP grade copper and the phase diagram of Cu-O it may be stated that in the range of low oxygen content (up to 0.008 wt.%) a variable solubility of oxygen in copper occurs. Further analysis of the diagram shows that at 1066 °C and up to 0.39 wt.% of oxygen the formation of Cu-Cu_2_O eutectic may be observed [14]. Regarding copper of higher oxygen content and the temperature up to 1200 °C the formation of CuO copper oxide occurs [15]. However, for electrical purposes the oxygen content in copper strands is usually between 150 ppm and 200 ppm which disqualifies the possibility of CuO oxide formation. During crystallization of copper with low oxygen content the structure of the casted material contains of copper and eutectics located at the grain boundaries, and during the solidification of the strand Cu_2_O oxide segregates into interdendritic spaces [16,17]. The adhesion strength of the metal oxide effectively determines the wettability of the metal-oxide phase boundary, therefore, the Cu_2_O oxide is poorly wettable by copper, which should be expressed with low level of contact forces of Cu_2_O particle—copper matrix [18,19]. According to [20,21] the Cu-Cu_2_O eutectic present in copper containing oxygen deteriorates the mechanical properties and its amount limitation during casting is recommended. The authors in [22,23] stated that Cu-Cu_2_O eutectic forming along the grain boundaries in the casted strand may be a stress concentrator, which might lead to cracks at later stages of processing. Numerous gassings and porosities occurring in the strand when combined with Cu_2_O oxide constitute areas of increased risk of prospective material continuity loss and may also adversely affect the properties of copper such as electrical conductivity [24,25], however, the presence of Cu_2_O oxide may be beneficial in terms of e.g., materials hardness [26]. One of the conclusions made in [27] is that the presence of Cu-Cu_2_O eutectic may also influence the type of fracture during tensile testing. However, oxygen presence in the copper structure has a numerous beneficial functions. A carefully controlled amount of oxygen throughout the manufacturing process of copper reduces the negative impact of impurities as it is involved in the formation of oxides or conglomerates of oxides of other less noble than copper elements, and therefore reducing their negative impact on material properties such as ductility and annealing by removing them from the solid solution into the form of precipitates [28,29]. The abovementioned matters have been included, among others, in the current paper, which analyses the samples of ETP grade copper taken parallelly and perpendicularly to the dendritic boundaries and the location of Cu_2_O oxide at the fracture surface. The novelty of the conducted research in the current paper was also the determination of Cu_2_O oxide location in the casted strand and its coherence with the copper matrix. The mean shape and size of the oxide was specified as well. The identification of the initial size of the oxide will allow to analyse its evolution in further plastic working processes including but not limited to hot rolling (wire rod manufacturing) and wire drawing process of ETP grade copper. The latter being a cold working process is characterized with significant level of strengthening of copper matrix and high unit pressure value on the wall of the die approach angle.

## 2. Experimental Procedures

### 2.1. Characteristics of the Research Material and SEM Observations 

An ETP grade copper strand with a cross section of 7200 mm^2^ (60 × 120 mm) obtained via continuous casting process with average amount of oxygen equal to 200 ppm has been tested. The casted strand was obtained with the defined casting parameters which were collectively presented in Table 1.

**Figure 1:**
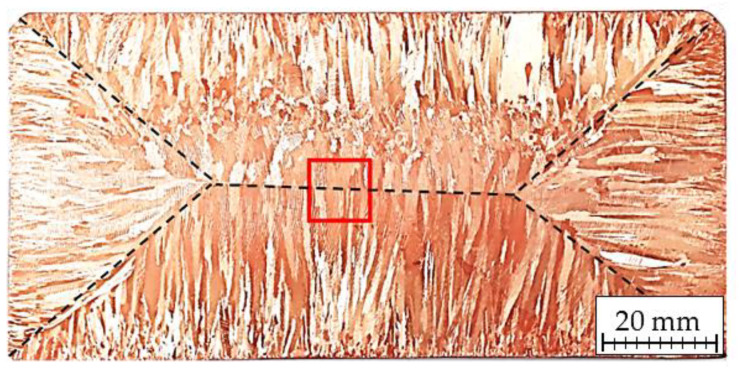
Macroscopic image of the tested ETP grade copper strand with marked area of the conducted light microscope analysis.

The strand was cut crosswise to the casting direction in several slices with thickness of 10 mm and was subjected to material properties analysis, in particular, macrostructural studies and micro-analysis of its chemical composition. Microstructural analysis of the copper strand using light microscope (Carl Zeiss. AG, Oberkochen, Germany) was also conducted with specific emphasis on the observations of Cu_2_O oxide morphology and the type of Cu-Cu_2_O boundaries. The macrostructure of the copper strand is presented at Figure 1 with the structural analysis area marked with a red square and black dashed lines showing the crystallization fronts formed during the strand casting process. The investigation of longitudinal sections, cross sections and fractures of ETP grade copper strand after uniaxial tensile test was conducted using a scanning electron microscope (SEM) model S-3500N (Hitachi Ltd., Tokyo, Japan), which is equipped with energy-dispersive X-ray microanalyzer (EDX) with which the studies of inclusions (Cu_2_O oxides) were performed and the spectra of characteristic X-rays were obtained.

### 2.2. Analysis of the Homogeneity of Electrical Properties of the Copper Strand

Electrical conductivity test was carried out in order to determine the homogeneity of the structure and the correctness of the copper strand casting process. Conductivity measurements were conducted at a grid of 72 equal fields with dimensions of 10 × 10 mm marked at the cross section of the strand using SigmaTest model 2.069 (Forester Instruments Inc., Pittsburgh, PA, USA), which is an eddy current device allowing accurate, non-destructive measurements of non-ferrous metals electrical conductivity based on the impedance of a measuring probe i.e., the relation between the voltage drop on the measured impedance and the current flowing in alternating current circuits. When measuring an unknown material the device converts the complex impedance value into an electrical conductivity value given in MS/m with a measuring range of the instrument determined by the calibration. The test was carried out at a constant ambient temperature of 20 °C. Absolute accuracy of the device is equal to ±0.5% of the measured value and the resolution is ±0.1% of measured value at 60 kHz frequency. Each of the 72 areas was measured three times and two different slices with 10 mm thickness were tested.

### 2.3. Analysis of the Mechanical Properties of the Casted Copper Strand

**Figure 2:**
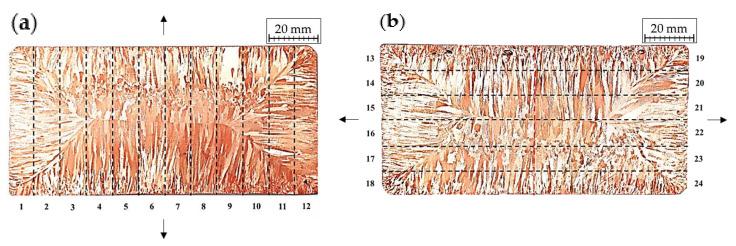
Schematics of division of copper strand into samples designated for uniaxial tensile test, where (**a**) the tensile force is parallel to the height of the strand, (**b**) the tensile force is perpendicular to the height of the strand.

The strands’ mechanical properties were assessed in the uniaxial tensile test using ProLine Z020 testing machine (Zwick/Roell Group, Ulm, Germany) and the obtained values of UTS, YS, and elongation were in detail analysed in terms of their relation with the fracture observations concerning the morphology and concentration of Cu_2_O oxides. Figure 2 shows the division of the copper strand on the samples designated for the uniaxial tensile test. Each of the samples was cut in the shape of 60 mm long paddle with a gauge length of 30 mm, width of the gauge length equal to 10 mm and thickness of 6 mm resulting in an initial cross section (S_0_) equal to approximately 60 mm^2^. The marked arrows indicate the direction of the tensile force during uniaxial tensile test.

## 3. Results and Discussion

### 3.1. Structure Observations and Micro Analysis of the Chemical Composition

**Figure 3:**
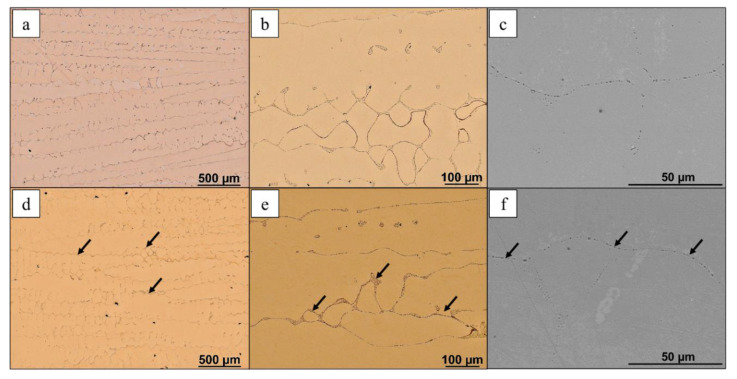
Cross section (**a**–**c**) and longitudinal section (**d**–**f**) of ETP grade copper strand. Light microscope, magnification (**a**,**d**) ×100, (**c**,**d**) ×500. SEM, secondary electron (SE), magnification (**e**,**f**) ×1000.

During solidification Cu_2_O oxide undergoes segregation and according to the observations in Figure 3 it is located in interdendritic spaces. The images show segregation of large oxide clusters at dendrite boundaries. The observed oxides are mainly located parallelly to the casting direction and as copper is a terrific thermal conductor and the solidification process is dynamic microsegregation takes place across most of the strand. In the tested material there is a discrete distribution of Cu_2_O monocrystals located in the spaces between grains and dendrites, which was confirmed with light microscopy images. Characteristic is the distribution of oxide, which form lines (paths) of discrete division of structure elements (dendrite arms, grains) or large clusters at the junction of several elements, which are flat images of spatially formed discrete clusters of oxide monocrystals and filling intracrystalline spaces/voids. The longitudinal section of the cast material, similarly to the cross section images, shows that the oxide crystallizes into large, regular clusters of monocrystals revealing various components of the copper matrix.

**Figure 4:**
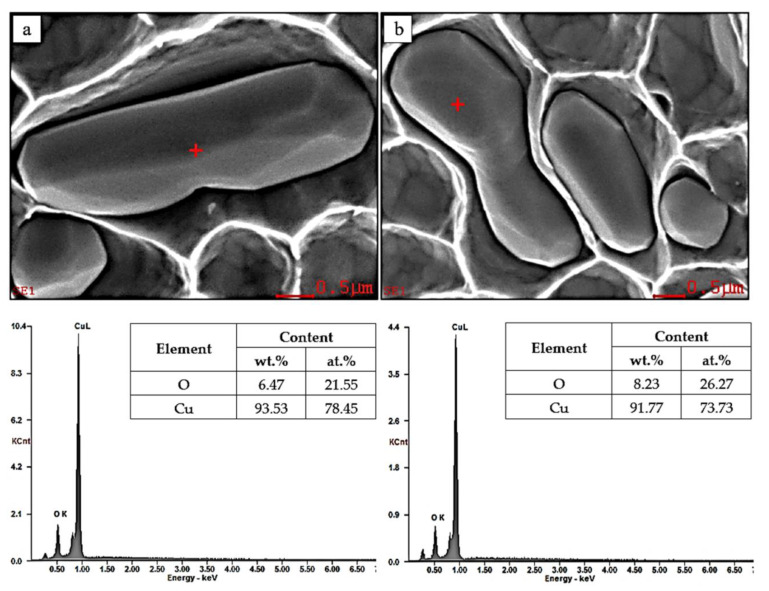
EDX analysis of the ETP grade copper strand with red cross marking the place of the chemical composition analysis and X-ray spectra of these points attached. SEM, SE, magnification ×10,000.

In order to accurately recognize and confirm Cu_2_O oxides inclusions an analysis of the microstructure was performed and the characteristic X-rays spectra were presented. Figure 4 shows EDX analysis of ETP grade copper strand with red cross marking the place of the chemical composition analysis and X-ray spectra of these points.

EDX analysis of the material did not show any other than presented Cu_2_O oxides inclusions. Each of the analysed particles of Cu_2_O confirmed the atomic ratio of oxygen to copper to be approximately 1:3, which is in agreement with stoichiometry of the Cu_2_O compound. The shape has been identified to be axi-symmetrical and oval with the size of approximately 2–5 μm.

### 3.2. Analysis of the Homogeneity of Electrical Properties of the Copper Strand

**Figure 5:**
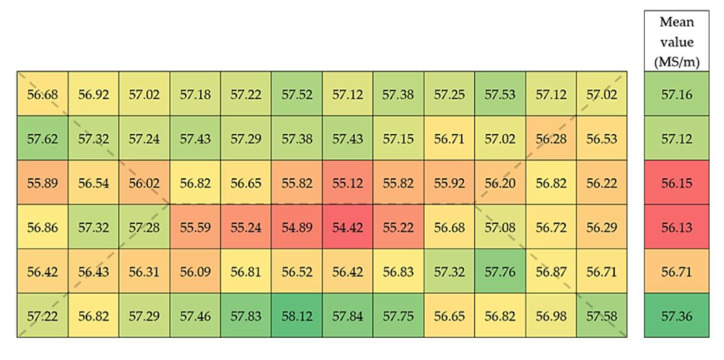
Electrical conductivity at the cross section of ETP grade copper strand with marked crystallization front.

Homogeneity of the structure and therefore the correctness of the strand casting process of copper was determined by electrical conductivity test using eddy current device. Figure 5 presents the results of electrical conductivity of the strand measured at the cross section. Electrical conductivity tests were carried out as an additional and complementing the structural images verification of the macrosegregation in the casted strand which was assumed due to the asymmetric structure of the strand resulting from the construction of the casting mould. For a more detailed analysis the mean values of electrical conductivity for the entire cross section and for each row were calculated.

The mean electrical conductivity of the material at the cross section was 56.77 MS/m with calculated standard deviation equal to 0.73. The tested material had similar electrical properties, which might indicate high homogeneity of the strand after the casting process. The crystallization front resulting from water cooling of the casted copper strand during the casting process is clearly visible at the cross section of the strand. Lower values of electrical conductivity being a consequence of oxygen macrosegregation at the axis of the crystallization fronts were recorded. It confirms the theoretical assumptions that at the place of contact of crystallization fronts the highest content of oxygen and interdendritic boundaries are present resulting in the reduction in electrical conductivity. Numerous accumulation of impurities in the material might lower its mechanical properties and may be the cause of materials discontinuity during its further processing.

### 3.3. Analysis of the Mechanical Properties of the Casted Copper Strand in Correlation with Structure and Presence of the Cu_2_O Oxides

Since Cu_2_O oxide is not wettable by copper causing the interfacial surfaces to be characterised with low mechanical strength, then in order to determine the places of Cu_2_O oxides crystallization and the bond strength of Cu-Cu_2_O at the interfacial surfaces and to define the mechanical properties of the copper strand a uniaxial tensile test was carried out using a testing machine with a maximum load of up to 2 tonnes. In order to perform the test from the prepared slices of strand samples were prepared as described in Section 2.3. The tensile force was applied in the parallel or perpendicular direction to the height (shorter side) of the strand as in Figure 2. Plasticity was determined based on the total elongation (A) of the samples and cross-sectional geometry of the sample. The plasticity of the material represented by the total elongation is usually determined with circular samples and when samples of different geometry are used e.g., square or rectangular the ratio of the cross sectional dimensions of the sample must be taken into account. In the current research paper, the plasticity investigation was conducted on samples with rectangular cross section of the gauge length. In the case of samples tested with tensile force parallel to the height (shorter side) of the strand it was assumed that at the location of the crystallization front the material would lose its cohesion and fracture at this area. However, in terms of the samples with tensile force applied perpendicularly to the height of the strand it was difficult to determine the outcome of the tensile test. Table 2 presents collectively the exact results concerning mechanical properties.

**Figure 6:**
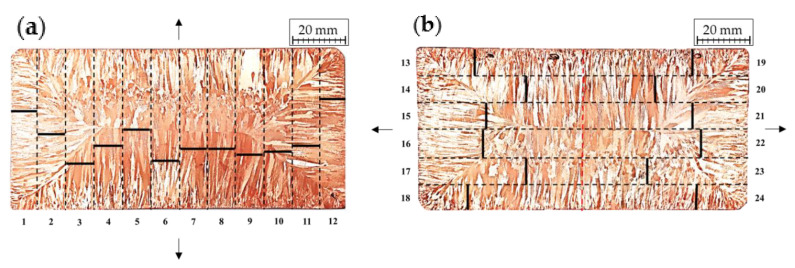
The places were fractures in uniaxial tensile test occurred, where (**a**) the tensile force is parallel to the height of the strand, (**b**) the tensile force is perpendicular to the height of the strand.

The study focused on determining the mechanical properties of the copper strand in uniaxial tensile strength did not show significant differences in terms of UTS regardless of the direction of the applied force. When considering YS it may be stated that the measured values are higher when the dendrite orientation and tensile force are parallel and the direction of the tensile force is parallel to the shorter side of the strand and the fractures of these samples were characterized as ductile. However, when the direction of the tensile force is perpendicular to the shorter side of the strand the YS values did not show significant differences. When considering total elongation, the values are visibly higher when the direction of the tensile force is parallel to the shorter side of the strand. The average UTS was approximately 163.9 MPa and the mean YS was equal to 46.1 MPa. The divergence in elongation values of the tested samples was between 7 and 27%. Sample marked as 13 had an internal defect that made it impossible to correctly read the given values. In order to determine the cause of the differences in total elongation values SEM analyses of the fractures were conducted and the places of fracture were marked at Figure 6 for both directions of the applied tensile force.

**Figure 7:**
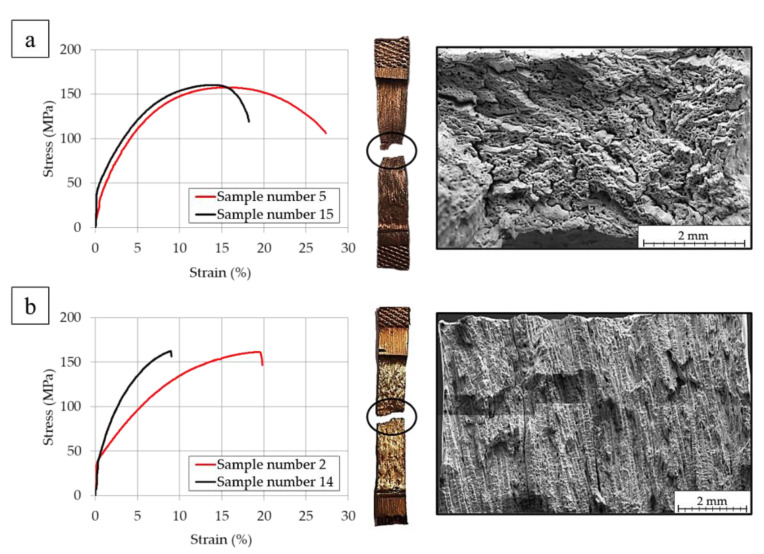
Stress-stress curves along with the images of the tested samples after uniaxial tensile test and a panoramic image of the fracture, where (**a**) samples number 5, 15 (**b**) samples number 2, 14 (according to Figure 6).

Only one of the tested samples ruptured at the crystallization front (sample marked as 5). Prior to the conducted tests it was assumed that the crystallization front would be a place where numerous impurities in the material would accumulate and therefore the material would lose its cohesion and fracture at this area. Additionally, in both of the analysed cases it is easily noticeable that the rupture points are fairly symmetrical. The analysis of the stress-strain curves shows that the material, depending on the grain orientation had two types of characteristic courses of the curve. Figure 7a depicts stress-strain curve of the material with crystallites arranged parallelly to the tensile force direction along with the image of the tested samples which is elongated with distinctive necking. However, in the case of the sample when the crystallites were arranged perpendicularly to the direction of the tensile force, a smaller scatter of YS results was observed (see Table 2) and smaller narrowing of the samples were observed as the samples were characterised with brittle fracture as in Figure 7b.

**Figure 8:**
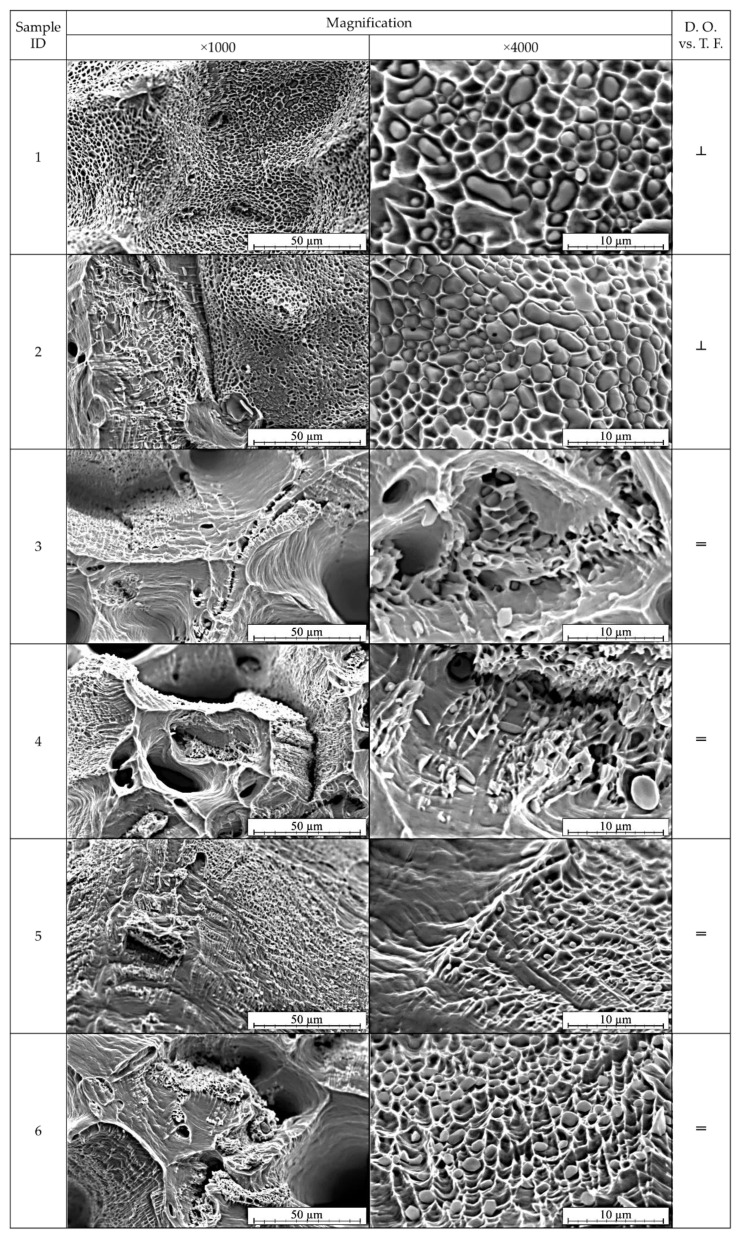
SEM analysis of the fractures after uniaxial tensile test of the samples prepared parallelly to the tensile force direction (see Figure 6). SEM, SE, magnification ×1000 and ×4000.

**Figure 9:**
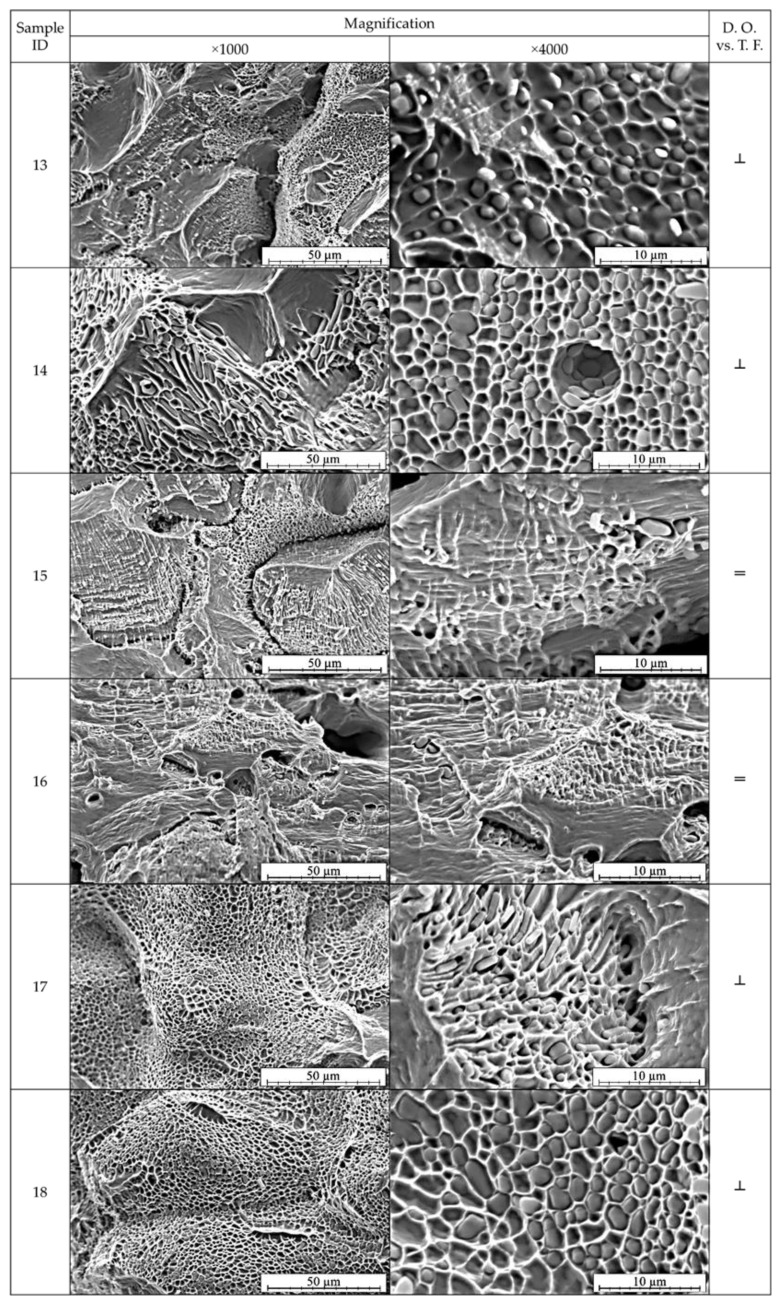
SEM analysis of the fractures after uniaxial tensile test of the samples prepared perpendicularly to the tensile force direction (see Figure 6). SEM, SE, magnification ×1000 and ×4000.

SEM analysis was conducted on the obtained fractures of the copper strand samples in order to determine the morphology and concentration of Cu_2_O oxides. Because of the observed and discussed previously symmetricity of the places where fractures occurred the analysis was carried out only for the first six samples in both parallel and perpendicular orientation of tensile force direction in relation to the shorter side of the strand. Figure 8 and Figure 9 depict the images of the fractures after the uniaxial tensile test in various magnifications.

It may be stated that the crystallization zones strongly affect the macroscopic plastic properties of the material. Based on the obtained images presented in Figure 8 and Figure 9 it is visible that the samples 1, 2, 13, 14, 17 and 18 were less ductile. The crystallites arranged perpendicularly to the direction of tensile force were the cause of lower mechanical properties of the strand and display anisotropy in terms of plastic properties revealed by the change in the sample cross section during the tensile test. The fractures did not show narrowing or the characteristic necking, whereas the higher magnification revealed that the fracture surface is filled with Cu_2_O oxide particles deeply embedded in the craters. In the case of samples marked as 3–6, 15 and 16 a higher ductile properties were observed as the crystallites were arranged parallelly to the direction of the tensile force. These results are in agreement with the studies conducted in [21,30,31]. Large narrowing of the tested samples was observed and with higher magnification the observed Cu_2_O oxides were shallowly located in the fracture craters, however, not as numerous as in the former analysed case. Clearly visible are the surfaces of division of copper matrix confirming the location of deformation and necking formation.

## 4. Conclusions

As a result of the conducted research concerning microstructure analysis, chemical composition analysis, mechanical and electrical properties tests it was found that a discrete distribution of Cu_2_O oxides on the surfaces of dendrites (in interdendritic spaces) is present in the copper strand.

Cu_2_O oxides are not wettable by copper and therefore the interfacial surfaces are characterised with low strength properties. The analysis of own research conducted in this paper shows that the crystallographic orientation of the structure (dendrite orientation) has a huge impact on the behaviour of copper during deformation in the uniaxial tensile test.

Regardless of the direction of the tensile force and orientation of the dendrites there were no visible changes in UTS values. However, when YS is considered it was confirmed that the measured values were higher when dendrite orientation and tensile force were aligned parallelly and the direction of the tensile force was parallel to the shorter side of the strand. The observed fractures of these samples were visibly ductile. On the other hand, when the direction of the tensile force was perpendicular to the shorter side of the strand the YS values did not show significant changes. Total elongation values were also higher when the direction of the tensile force was parallel to the shorter side of the strand.

The location of the Cu_2_O oxide particles is characteristic for specific fractures. Concerning brittle fractures, numerous oxides were located at the bottom of the craters while in the case of ductile fractures, the oxides were located shallowly (the oxides were pulled out of the copper matrix).

## Figures and Tables

**Table 1:** Continuous casting parameters of ETP grade copper strand.

Parameter	Cooling Water Pressure	Casting Temperature	Casted Strand Temperature	CASTING FEED	Protective Environment
Value	0.6 MPa	1115 °C	900 °C	11 m/min	Not present

**Table 2:** Mechanical properties of the copper strand samples subjected to uniaxial tensile test.

Sample Number	Direction of the Tensile Force	S_0_	YS	UTS	A	Dendrite Orientation vs. Tensile Force (D. O. vs. T. F.)	Fracture Characterisation
		mm^2^	MPa	MPa	%		
1	Parallel to the shorter side of the strand	58.5	40.2	162.3	17.5	┴	Brittle
2		59.0	40.8	161.2	19.5	┴	Brittle
3		59.5	45.5	155.4	21.1	═	Ductile
4		59.6	46.1	157.2	26.1	═	Ductile
5		60.0	46.8	157.5	26.8	═	Ductile
6		59.2	54.2	164.5	22.2	═	Ductile
7		59.0	52.6	161.2	15.1	═	Ductile
8		58.9	51.1	159.8	16.2	═	Ductile
9		56.8	48.4	165.8	25.4	═	Ductile
10		60.4	45.6	164.4	20.8	═	Ductile
11		60.1	40.7	168.6	16.1	┴	Brittle
12		59.9	40.4	169.1	18.4	┴	Brittle
13	Perpendicular to the shorter side of the strand	59.4	- *	- *	- *	┴	Brittle
14		58.5	48.1	162.4	8.6	┴	Brittle
15		59.4	46.9	160.3	18.1	═	Ductile
16		59.5	47.1	169.4	17.6	═	Ductile
17		60.0	44.2	170.1	14.9	┴	Brittle
18		59.0	47.2	168.5	8.9	┴	Brittle
19		58.6	45.6	153.6	7.1	┴	Brittle
20		58.9	45	164.8	14.3	┴	Brittle
21		58.9	44.1	161.2	12.3	═	Ductile
22		60.2	46.8	174.1	10.2	═	Ductile
23		61.0	45.4	175.8	8.1	┴	Brittle
24		60.1	48.3	160.6	9.2	┴	Brittle

* material with internal defect.
